# Variable effects of wolves on niche breadth and density of intraguild competitors

**DOI:** 10.1002/ece3.8542

**Published:** 2022-02-09

**Authors:** Nicholas L. Fowler, Tyler R. Petroelje, Todd M. Kautz, Nathan J. Svoboda, Jared F. Duquette, Kenneth F. Kellner, Dean E. Beyer, Jerrold L. Belant

**Affiliations:** ^1^ Global Wildlife Conservation Center College of Environmental Science and Forestry State University of New York Syracuse New York USA; ^2^ Alaska Department of Fish and Game, Kodiak Kodiak Alaska USA; ^3^ Division of Wildlife Resources Illinois Department of Natural Resources Champaign Illinois USA; ^4^ Wildlife Division Michigan Department of Natural Resources Marquette Michigan USA

**Keywords:** bobcats, carnivore, competition, coyotes, niche variation hypothesis, realized niche

## Abstract

1. The parallel niche release hypothesis (PNR) indicates that reduced competition with dominant competitors results in greater density and niche breadth of subordinate competitors and which may support an adaptive advantage.
2. We assessed support for the PNR by evaluating relationships between variation in niche breadth and intra‐ and interspecific density (an index of competition) of wolves (*Canis lupus*) coyotes (*C. latrans*), and bobcats (*Lynx rufus*).
3. We estimated population density (wolf track surveys, coyote howl surveys, and bobcat hair snare surveys) and variability in space use (50% core autocorrelated kernel density home range estimators), temporal activity (hourly and overnight speed), and dietary (isotopic δ^13^C and δ^15^N) niche breadth of each species across three areas of varying wolf density in the Upper Peninsula of Michigan, USA, 2010–2019.
4. Densities of wolves and coyotes were inversely related, and increased variability in space use, temporal activity, and dietary niche breadth of coyotes was associated with increased coyote density and decreased wolf density supporting the PNR. Variability in space use and temporal activity of wolves and dietary niche breadth of bobcats also increased with increased intraspecific density supporting the PNR.
5. Through demonstrating decreased competition between wolves and coyotes and increased coyote niche breadth and density, our study provides multidimensional support for the PNR. Knowledge of the relationship between niche breadth and population density can inform our understanding of the role of competition in shaping the realized niche of species.

## Graphical abstract

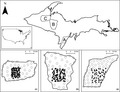

## 1 INTRODUCTION

The realized niche of a population encompasses the ecological conditions which facilitate persistence when individuals are constrained by competition (Case & Gilpin, 1974). In carnivores, competition may be direct (e.g., interference and predation) or indirect (e.g., exploitative and trophic cascades) and mediates relationships among species (Hunter & Caro, 2008). Release from competition with dominant species may then allow for broadening of niche dimensions in either the niche width across all individuals in the population (parallel release hypothesis) (Bolnick et al., 2010) or through increased among‐individual variation (i.e., the niche variation hypothesis) (Van Valen, 1965). Broadly, increased population niche width is suspected to support an adaptive advantage for populations (Costa et al., 2008), though empirical evidence linking niche width to an index of fitness is rare. Conversely, reduced intraspecific competition of subordinate carnivores through decreased population density may also result in reduced variation in diet, space use, or temporal activity, due to increased competition with dominant carnivores (Manlick et al., 2017). Behavioral adjustments reducing niche overlap facilitate species co‐existence under the competitive exclusion principle (Gause, 1932).

Interspecific competition among carnivores is often presumed to manifest as inverse density relationships between species (Ripple et al., 2013). With decreased interspecific competition, high population densities of subordinate carnivores may reflect a population with sufficient resources and fewer constraints (MacArthur et al., 1972; Codron et al., 2018). Concurrently, estimating niche breadth and density of competitors allows for estimation of the degree of competition among and within species (e.g., Berger & Gese, 2007; Jesmer et al., 2020) and the detection of competition‐induced niche variability (e.g., Lafferty et al., 2015; Novosolov et al., 2018). Knowledge of the relationship between niche breadth and population density can inform our understanding of the role of intra‐ and interspecific competition in shaping the realized niche of species (Maguire, 1973; Sibly & Hone, 2002).

Extirpation of dominant carnivores (e.g., wolves [*Canis lupus* and *C. rufus*], brown bears [*Ursus arctos*], and mountain lions [*Puma concolor*]) from much of their historical ranges during the 18th–19th centuries resulted in increased abundances and distributions of subordinate carnivores (e.g., coyotes [*C. latrans*], foxes [*Vulpes* spp.], and bobcats [*Lynx rufus*]) released from competition (Prugh et al., 2009). Functioning at higher trophic levels in reduced trophic webs, expansion of subordinate carnivores can alter interspecific competition and predator–prey relationships (Ripple et al., 2013). Increased density of subordinate carnivores has led to declines in prey species (Kilgo et al., 2012; Levi & Wilmers, 2012) and competitors (Levi & Wilmers, 2012). Following increased protections and reintroductions, dominant carnivores have recolonized portions of their ranges (Gompper et al., 2015) and are now sympatric with subordinate carnivores which historically occurred at lower densities or were absent (Arjo & Pletscher, 2000; Mech, 1995; Swenson et al., 2000). Recolonization of dominant carnivores has led to extirpations (Peterson, 1995), reduced densities (Berger & Gese, 2007), and behavioral adjustments (Arjo & Pletscher, 2000) of subordinate species and influenced prey populations (Estes et al., 2011). However, investigations of the role of dominant carnivores limiting subordinate carnivores rarely consider space use, temporal activity, and diet concurrently (e.g., Berger & Gese, 2007; Santos et al., 2007; Schuette et al., 2013; Smith et al., 2018) or infer niche effects solely through population estimation (e.g., Levi & Wilmers, 2012; Ripple & Beschta, 2006). Additionally, indirect effects of dominant carnivores on species which may be subordinate to multiple species (e.g., bobcats potentially subordinate to coyotes and wolves) are poorly understood (Ripple et al., 2013).

Following natural recolonization, gray wolf populations have stabilized in the Upper Peninsula of Michigan (the UP) since 2011 (Michigan Department of Natural Resources [MDNR], 2015). Utilizing data from the concurrent Michigan Predator Prey Project, we evaluated three areas of reported varying wolf density to evaluate our hypothesis that where competition with wolves (and coyotes for bobcats) is reduced, increased intraspecific competition for all species results in broader population level niche breadth through parallel release and an adaptive advantage indexed by greater subordinate species density. We predicted coyote and wolf niche breadth and population density would be inversely related. Coinciding with increased wolf density, we predicted increased niche breadth and density of bobcats due to decreased competition with coyotes and increased bobcat density. We tested our predictions by evaluating variability in space use, temporal activity, and diet.

## 2 STUDY AREA

**FIGURE 1:**
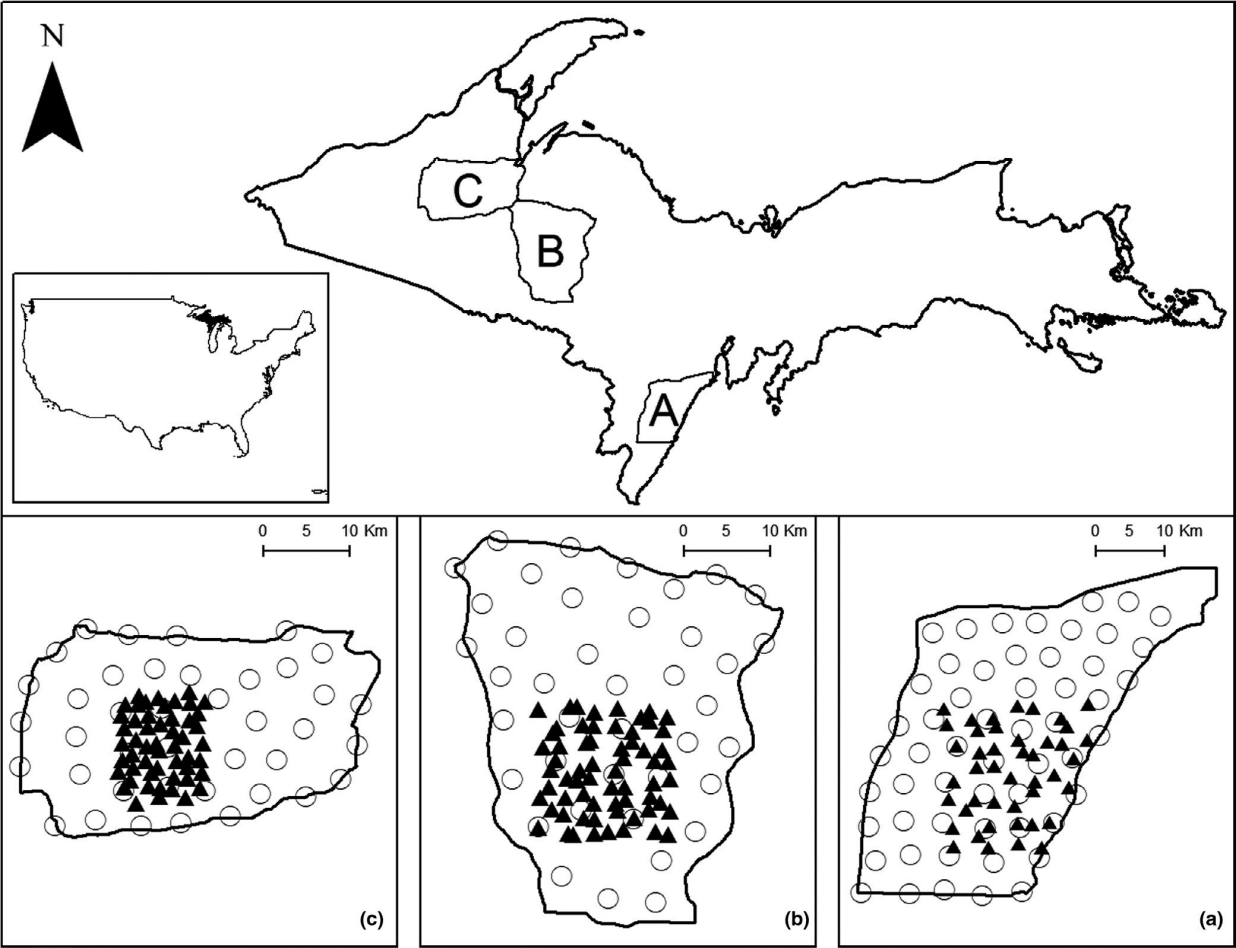
Locations of study areas (ESC, CF, and SM) used to assess patterns in niche variability of gray wolves (*Canis lupus*), coyotes (*C*. *latrans*) and bobcats (*Lynx rufus*), Upper Peninsula of Michigan, USA, 2010–2019. Symbols within insets represent bobcat hair snare sites (black triangles) and coyote howls survey sites (open circles)

We conducted the study across three areas in the UP (Figure 1, Table S1). We collected data from the Escanaba study area (ESC; 871 km^2^) during 2010–2011 (45.74–45.40°, −87.61 to −87.08°). Most of ESC was forested woody lowland (52%) with other dominant land covers including deciduous hardwood forests and pastures (2011 National Land Cover Data, Homer et al., 2015). Dominant tree species included eastern white cedar (*Thuja occidentalis*), eastern hemlock (*Tsuga Canadensis*), balsam fir (*Abies balsamea*), pine (*Pinus* spp.), trembling aspen (*Populus tremuloides*), and sugar maple (*Acer saacharum*). Primary land ownership included commercial forest association and privately held lands (60%) and state land (38%). Number of human residents within the study area was 9741; and combined density of permanent and seasonal housing was 6.65 per km^2^ (US Census Bureau, 2010). Monthly temperatures ranged from average highs of 24.4°C during July to average lows of −10.9°C during January; and snowfall ranged from 50 to 150 cm (National Oceanic and Atmospheric Administration 1981–2010; https://www.ncdc.noaa.gov/cdo‐web/datatools/normals summary).

Data from the Crystal Falls study area (CF; 1831 km^2^) were collected during 2012–2015 (46.59–46.08°, −88.52 to –87.92°) (Figure 1, Table S1). Most of CF was forested (86%) with dominant land covers including deciduous hardwood forests, mixed forests, and woody wetlands (Homer et al., 2015). Dominant tree species included sugar maple, trembling aspen, black spruce (*Picea mariana*), and red pine (*Pinus resinosa*). Primary land ownership consisted of Commercial Forest Association and private lands (82%) and state land (18%). Number of human residents within the study area was 4776, and combined density of permanent and seasonal housing was 3.21 per km^2^ (US Census Bureau, 2010). Monthly temperatures ranged from average highs of 25.8°C during July to average lows of −12.0°C during January, and snowfall ranges from 125 to 250 cm (National Oceanic and Atmospheric Administration 1981–2010; https://www.ncdc.noaa.gov/cdo‐web/datatools/normals summary).

Data from the Silver Mountain study area (SM; 1543 km^2^) were collected during 2016–2019 (46.78–46.47°, −89.18 to −88.43°) (Figure 1, Table S1). Dominant land covers included deciduous, evergreen, and mixed forests with less lowland forest than in CF (Homer et al., 2015). Dominant tree species included sugar maple, eastern white pine (*Pinus strobus*), trembling aspen, eastern hemlock, black spruce, and northern white cedar. Primary land ownership consisted of federal and state land (83%). Number of human residents within the study area was 4389 and combined density of permanent and seasonal housing was 1.99 per km^2^ (US Census Bureau, 2010). Monthly temperatures ranged from average highs of 24.8°C during July to average lows of −17.8°C during January; snowfall ranges from 225 to 400 cm (National Oceanic and Atmospheric Administration 1981–2010; https://www.ncdc.noaa.gov/cdo‐web/datatools/normals summary).

## 3 METHODS

### 3.1 Population estimation

We estimated wolf density annually during January–March in each study area using repeated track surveys (Table S1; Beyer et al., 2009). Surveys were generally conducted 12–72 h after snowfall to allow for wolf movement and limit deterioration of tracks. We traveled using on‐ and off‐road vehicles on roads and trails to locate tracks which were followed until the number of individuals traveling together could be determined. Packs were surveyed on >3 occasions each winter. We used spatial data from collared individuals to estimate pack boundaries and packs were repeatedly surveyed to estimate the minimum number of individuals per pack (Beyer et al., 2009).

We estimated coyote density during July–October via occupancy modeling using howl surveys at 55, 40, and 40 sites in ESC, CF, and SM, respectively (Figure 1, Table S1). We established survey sites along roads and completed eight survey occasions at 10‐day intervals. We assumed a 2 km detection radius and buffered sites by >4 km to avoid double counting during the same occasion (Petroelje et al., 2013). At each survey site, we broadcasted prerecorded coyote‐group‐yip howls using a FX3 game caller (FoxPro, Lewiston, Pennsylvania, USA) and recorded detections of ≥1 coyotes. Surveys were not conducted when wind speeds were ≥12.8 km per hour or during precipitation to increase detection (Harrington & Mech, 1982). From the detection data, we fit intercept‐only abundance mixture models and followed sampling and statistical analyses described by Petroelje et al. (2013) in ESC.

In each study area, we estimated bobcat density using open population spatial capture–recapture modeling (Gardner et al., 2010; Kautz et al., 2019; Whittington & Sawaya, 2015). We sampled bobcats using break‐away hair snares (Stricker et al., 2012) at 44, 64, and 52 sites within grid cells of 2.5, 6.25, and 5.0 km^2^ in ESC, CF, and SM, respectively (Figure 1, Table S1). Sampling sites were placed in preferred winter bobcat habitat of lowland coniferous habitats and in riparian areas to increase encounters (Lovallo & Anderson, 1996). Sites were baited using partial white‐tailed deer (*Odocoileus virginianus*) or beaver (*Castor canadensis*) carcasses with commercial trapping lures placed 2 m above ground. Surveys were performed for eight occasions at 7‐day intervals during January–March; and sites were re‐baited and lured every 7 days as needed. However, extreme winter weather limited the final bobcat hair snare to six sampling occasions in the SM. Wildlife Genetics International (Nelson, British Columbia, Canada) performed genotyping and laboratory techniques, sampling processing, and population modeling followed methods performed by Stricker et al. (2012) in ESC and Kautz et al. (2019) in CF.

### 3.2 Capture and sample collection

We captured coyotes, bobcats, and wolves during May–July using foothold traps (No. 3 soft catch; Oneida Victor, Cleveland, Ohio, USA or MB‐750; Minnesota Trapline Products, Inc.) (Table S1). Coyotes also were captured using relaxing locking cable restraints (Wegan et al., 2014) during February–March (Petroelje et al., 2014). Bobcats also were captured during March–April using cage traps (Norm Blackwell Cat Collector and HAVAHART Collapsible traps) baited with white‐tailed deer and beaver remains and commercial lures. Anesthetization, collaring, and reversal of immobilizing agents followed Petroelje et al. (2013), Petroelje et al. (2019) and Svoboda et al. (2013) for coyotes, wolves, and bobcats, respectively. Carnivores were fitted with a global positioning system (GPS) collar with a very high‐frequency (VHF) transmitter (Model GPS 7000SU and LiteTrack 330, Lotek Wireless Inc. Newmarket Ontario, Canada and VERTEX PLUS Vectronic Aerospace GmbH, Berlin, Germany [various belting and battery size configurations based on animal size]). We programmed collars to collect locations every 15 min May 1–August 31. We performed data uploads using ultra high frequency communication to a handheld command unit (Lotek Wireless Inc. Newmarket, Ontario, Canada) from an aircraft. Capture and handling procedures were approved by Mississippi State University and State University of New York, College of Environmental Science and Forestry Institutional Animal Care and Use Committees (protocols: #09‐004, #12‐012, #15‐013, #17‐119, #180501).

### 3.3 Space use

We included individuals with >10 days of consecutive data during June 1–August 31, excluded relocations ≤5 days of capture (Brivio et al., 2015), and excluded individuals without discernable ranges (Noonan et al., 2019). We fit continuous time movement models via maximum likelihood and calculated autocorrelated kernel density home range estimators (AKDE) and estimated 50% kernel density core utilization distributions (Calabrese et al., 2016) to represent core ranges (Finnegan et al., 2021). The AKDE technique allows for home range estimation while accounting for serial autocorrelation which is inherent in traditional kernel density estimators (Calabrese et al., 2016; Fleming et al., 2014, Fleming & Calabrese, 2017). We compared intrapopulation variability in home range size via the population variance and Bartlett's K‐squared (B‐K^2^) test for equal variances (*α* = 0.05) which is robust to unequal sample sizes among groups (Bartlett, 1937; Marwick & Krishnamoorthy, 2019).

### 3.4 Activity

We estimated mean hourly speed (meters per second) of individuals within species and study areas from consecutive relocations which occurred within 14–16 min of each other from consecutive relocation collar data. We evaluated intrapopulation variability in temporal activity with increased wolf density using one‐tailed B‐K^2^ tests for equal variances (Bartlett, 1937; Marwick & Krishnamoorthy, 2019) for each hour (*α* = 0.05). We then quantified total intrapopulation variability in temporal activity as population variance of overnight (22:00–06:00) speed (meters per second) which is when carnivores were most active in our study.

### 3.5 Diet

While animals were anesthetized, we collected about 15 guard hairs from between the scapulae. Whole hair samples were stored in paper envelopes, prepared by the MDNR Wildlife Disease Laboratory, and analyzed at the Idaho State University Stable Isotope. Hair samples were cleaned by immersion in methanol and chloroform (2:1 v/v), rinsed in distilled water, and dried at 60°C. Homogenized samples were analyzed using an elemental analyzer coupled with a Finnigan Delta plus isotope ratio mass spectrometer for δ^13^C and δ^15^N measurements. All isotope values are reported in per mil units (‰) according to the relationship δ*X* = [(*R*sample/*R*standard) − 1] × 1000‰, where *X* is the element of interest, and *R* is the measured isotopic ratio. All carbon isotope measurements are relative to the Vienna Peedee Belemnite standard, and all nitrogen measurements are relative to atmospheric nitrogen. As wolves (Darimont & Reimchen, 2002), coyotes (Bekoff & Gese, 2003), and bobcats (Warsen et al., 2014) synthesize hair during autumn, results represent assimilated diet coinciding during November–February (Stains, 1979). We estimated population variability in dietary niche breadth by the convex hull of isotope axes for each species (Layman et al., 2007).

### 3.6 Niche breadth and density

We assessed the relationships between niche breadth and intra‐ and interspecific carnivore densities by study area using Pearson's product moment correlation coefficient (*r*) (Dormann et al., 2013). We scaled niche breadth and density estimates to 0–1 within species and considered coefficients ≥0.70 strongly correlated. We did not consider linear significance due to sensitivity of small sample size, and we relied on interpretation of relationships between niche breadth and intra‐ and interspecific densities along with directionality of strength of correlations.

## 4 RESULTS

### 4.1 Population estimation

**FIGURE 2:**
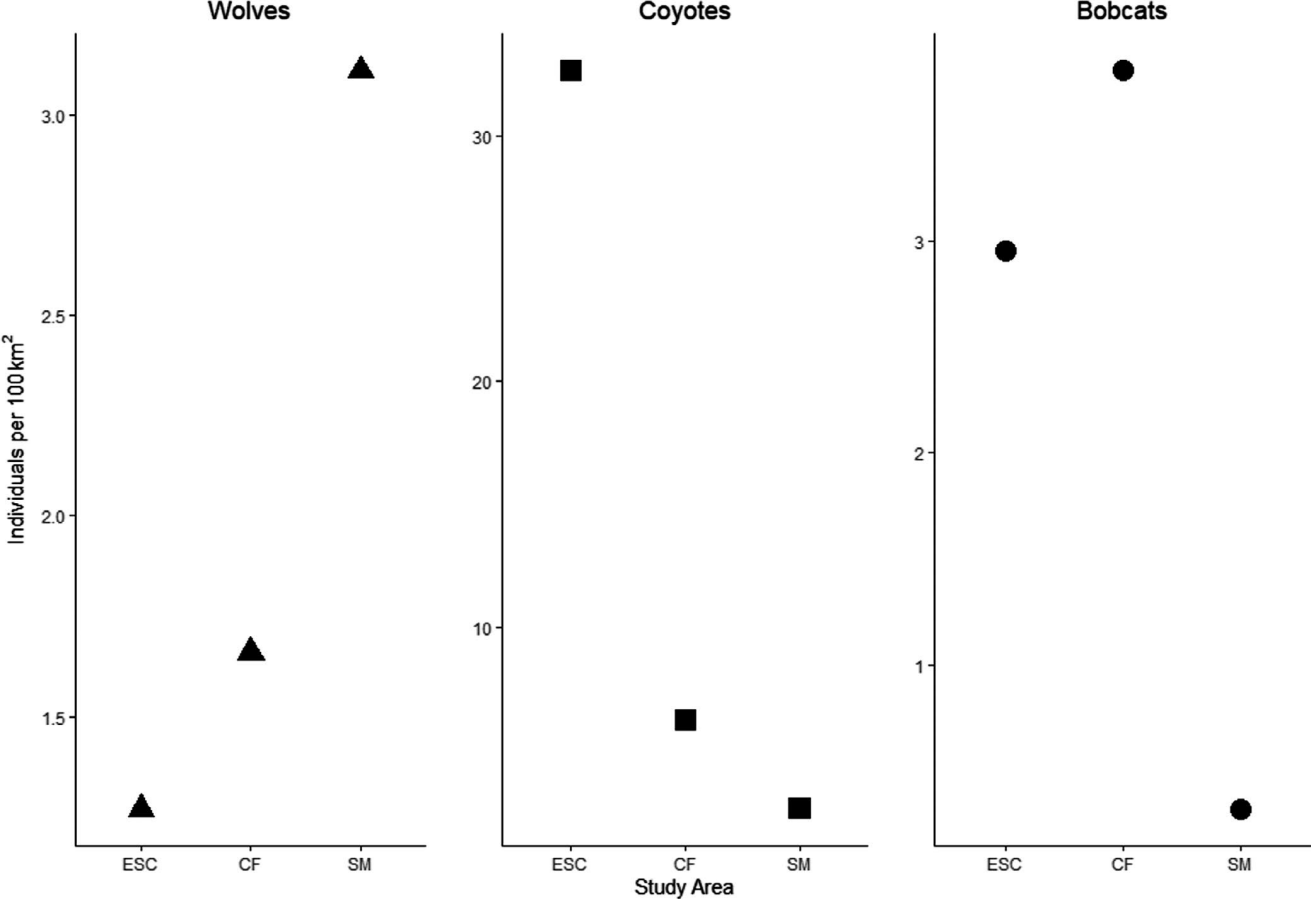
Density estimates of gray wolves (*Canis lupus*) (solid line), coyotes (*C*. *latrans*) (dashed line), and bobcats (*Lynx rufus*) (dotted line) across three study areas (ESC, CF, SM), Upper Peninsula of Michigan, USA, 2010–2019

We identified two, four, and eight wolf packs overlapping ESC, CF, and SM corresponding to estimated densities of 1.27, 1.66, and 3.1 individuals per 100 km^2^, respectively (Figure 2, Results S1, Table S3). Coyote density decreased 81.2% and 58.3% as wolf density increased and was negatively correlated with increased wolf density (*r* = −.74, Table 1). Due to few detections across years, we were unable to estimate bobcat density in SM and report density as the number of individuals detected through hair snares and captured across years at seven individuals (0.32 individuals per 100 km^2^). Bobcat density initially increased 22.4% from ESC to CF but then declined by 91.9% from CF to SM as wolf density increased (*r* = −.91); and coyote density decreased (*r* = .38) from ESC to CF to SM.

**TABLE 1:** Scaled niche breadth (0–1) and density of gray wolves (*Canis lupus*), coyotes (*C. latrans*), and bobcats (*Lynx rufus*) among three study areas, Upper Peninsula of Michigan, USA, 2010–2019

Species	Niche axis^a^	Study area		
		ESC	CF	SM
		Density		
Wolves	–	0.00	0.21	1.00
Coyotes	–	1.00	0.12	0.00
Bobcats	–	0.76	1.00	0.00

		Niche breadth		
Wolves	Spatial	0.00	0.18	1.00
	Temporal	0.00	0.17	1.00
	Dietary	1.00	0.00	0.98
Coyotes	Spatial	1.00	0.28	0.00
	Temporal	1.00	0.22	0.00
	Dietary	1.00	0.07	0.00
Bobcats	Spatial	1.00	0.00	0.20
	Temporal	0.00	1.00	0.90
	Dietary	1.00	0.69	0.00

^a^ Spatial = Population variance of autocorrelated utilization distribution estimates for core (50% kernel density) territories (km^2^); Temporal = Population variance of mean overnight speed (m/s); Dietary = Area of convex hull describing isotopic signatures (‰ δ^13^C and δ^15^N).

### 4.2 Space use

We identified 33 wolves (16 females and 17 males), 35 coyotes (21 females and 14 males), and 10 bobcats (three females and seven males) with data meeting our inclusion criteria (Table S2, Results S1). Ratio of collared individuals to mean annual study area density estimates were 53%, 69%, and 70% for wolves; 3%, 9%, and 8% for coyotes; and 6%, 8%, and 60% (density based on minimum detected) for bobcats in ESC, CF, and SM, respectively.

**FIGURE 3:**
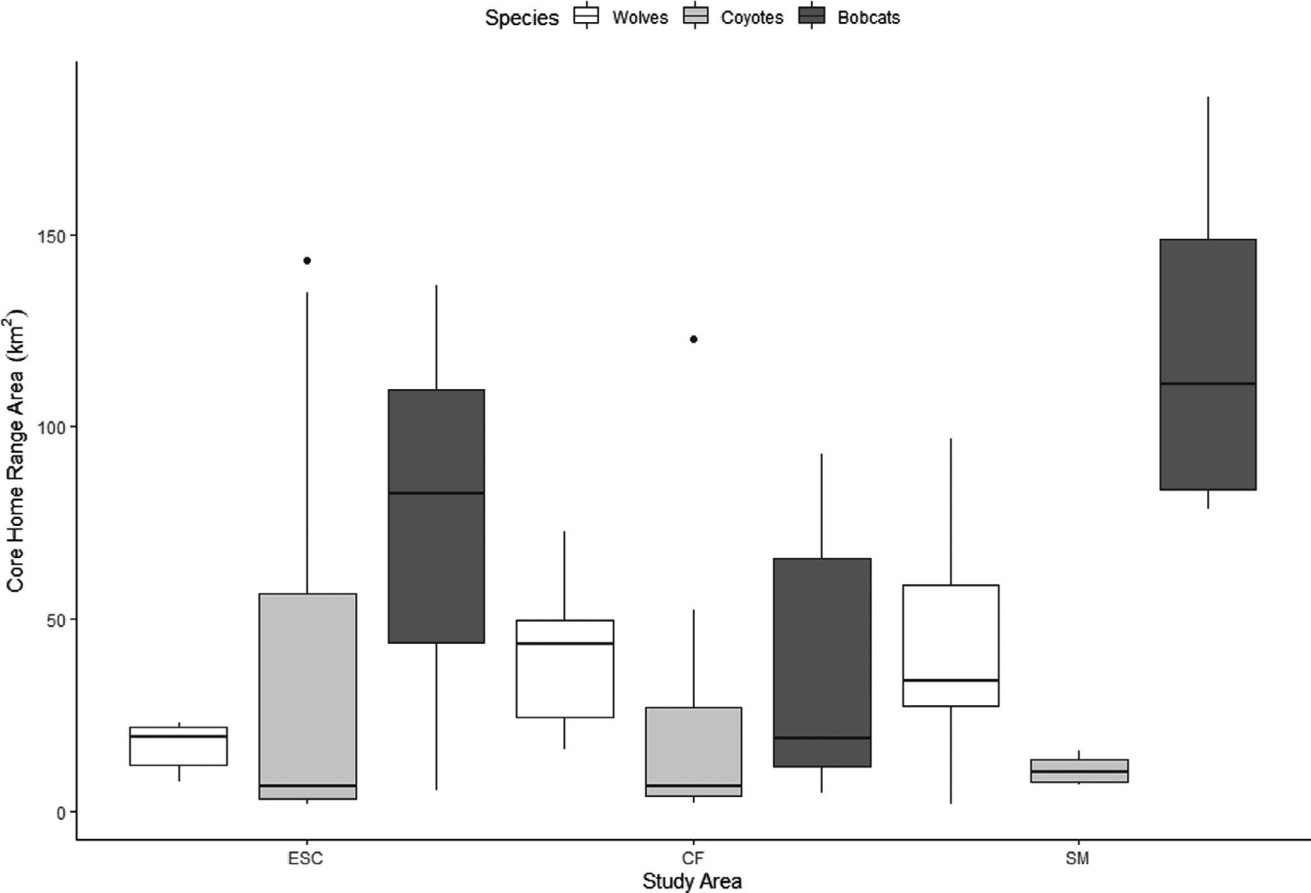
Boxplots comparing variability in autocorrelated kernel density estimates of core (50% kernel density) territories of wolves (*Canis lupus*), coyotes (*C. latrans*), and bobcats (*Lynx rufus*) across three study areas (ESC, CF, SM), Upper Peninsula of Michigan, USA, 2010–2019. Horizontal lines represent median, upper, and lower box bounds represent 75th and 25th percentile, respectively. Upper and lower limits of vertical lines represent largest and smallest value within 1.5 times interquartile range above and below the 75th and 25th percentile, respectively. Dots represent outliers > quartile 3 + 1.5 * interquartile range

Increased variability in wolf space use increased with increasing wolf density (ESC–CF: B‐K^2^ = 4.28, *p*‐value = .04; ESC–SM: B‐K^2^ = 7.42, *p*‐value < .01, Figures 2 and 3). Conversely, variability in coyote space use decreased with decreasing coyote and increasing wolf density (ESC–SM: B‐K^2^ = 10.48, *p*‐value < .01; CF–SM: B‐K^2^ = 8.67, *p*‐value < .01). Increased variability in wolf space use was also correlated with increasing wolf density (*r* = .99) (Table 1, Figure 3). Increased variability in coyote space use correlated with increased coyote density (*r* = .98) and decreased wolf density (*r* = −.84). Increased variability in space use of bobcats was uncorrelated with decreased bobcat (*r* = .10) and increased wolf density (*r* = −.51) but was correlated with decreased coyote density (*r* = .96).

### 4.3 Temporal activity

**FIGURE 4:**
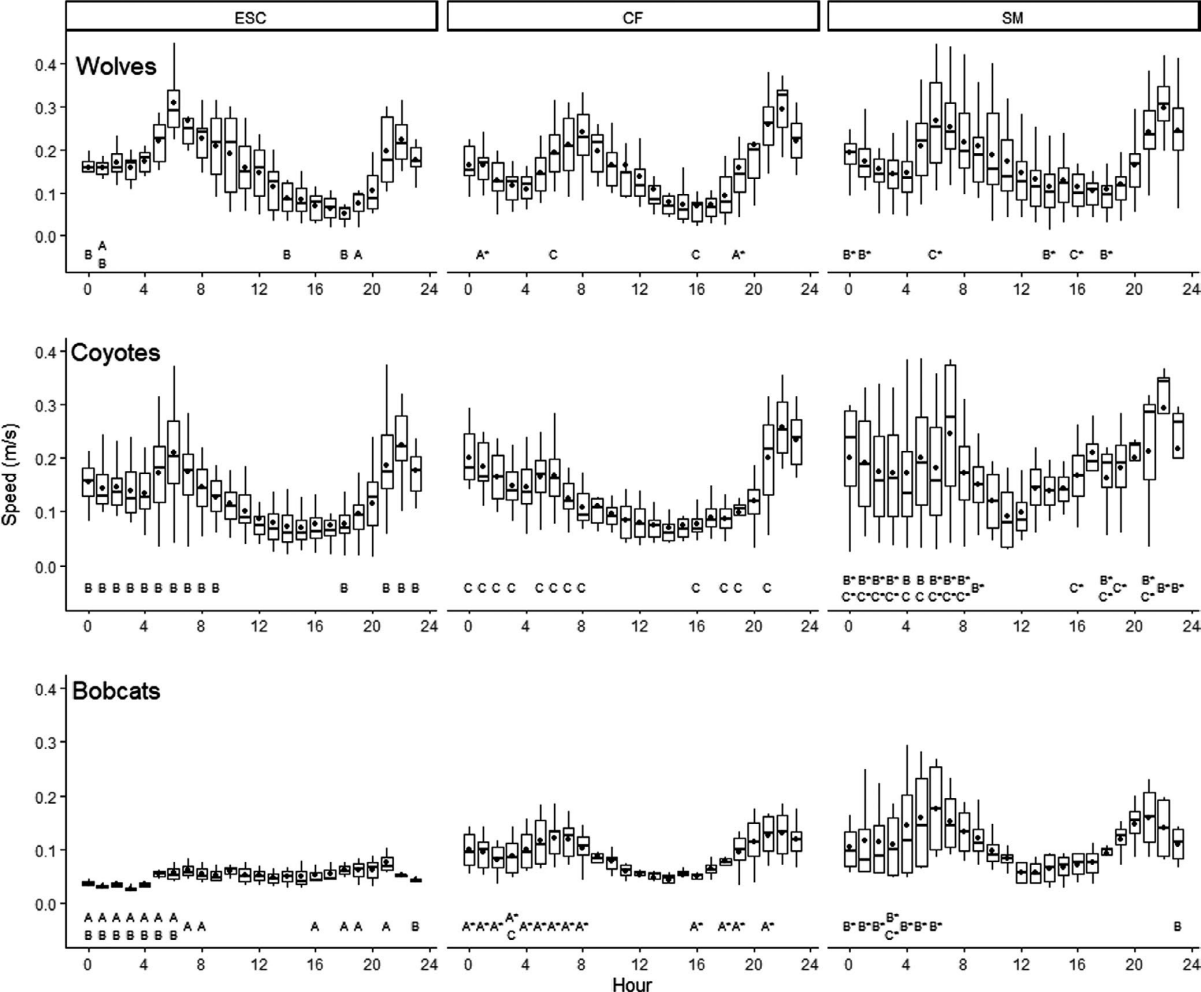
Boxplots of diel patterns in speed of wolves (*Canis lupus*), coyotes (*C. latrans*), and bobcats (*Lynx rufus*) across three study areas, Upper Peninsula of Michigan, USA, 2010–2019. Horizontal lines represent median, dots represent mean, upper, and lower box bounds represent 75th and 25th percentile, respectively. Upper and lower limits of vertical lines represent largest and smallest value within 1.5 times interquartile range above and below the 75th and 25th percentile, respectively. Hours with matching letters represent statistical significance (*p *< .05) and asterisk denotes larger variance among study areas using Bartlett's K‐squared test of homogeneity of variance

Variability in hourly activity of wolves increased haphazardly among diel hours with increased wolf density (Figures 2 and 4, Results S1). Variability in evening and overnight/early morning activity of coyotes and bobcats generally increased with decreased coyote density and increased wolf density. Increased variability in overnight activity of wolves was correlated with increased wolf density (*r* = .99, Table 1). Increased variability in overnight activity of coyotes was correlated with increased coyote density (*r* = .98) and decreased wolf density (−.80). Increased variability in overnight activity of bobcats was correlated with decreased coyote density (*r* = −.98), weakly to decreased wolf density (*r* = .59), but was uncorrelated to increased bobcat density (*r* = −.19).

### 4.4 Diet

**FIGURE 5:**
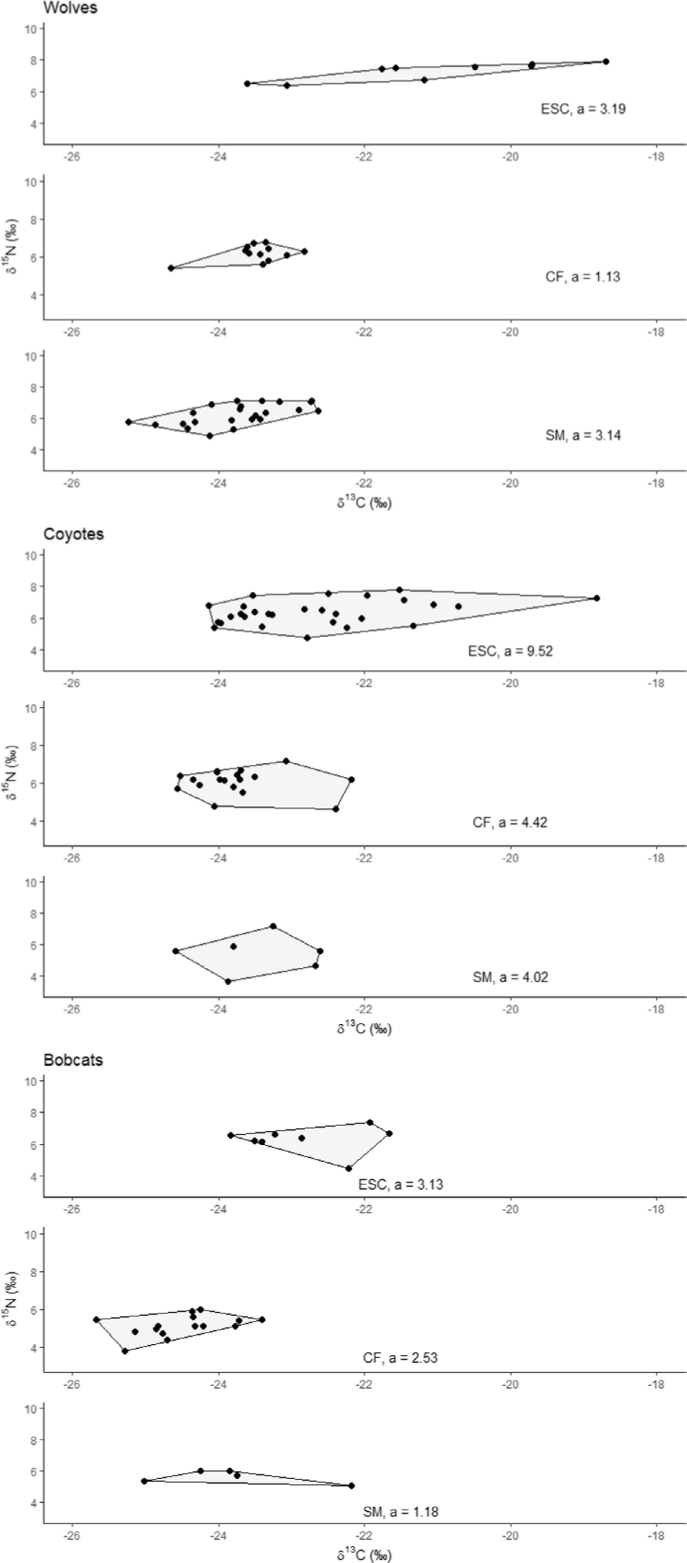
Isotopic signatures (δ^13^C and δ^15^N) of gray wolves (*Canis lupus*), coyotes (*C. latrans*), and bobcats (*Lynx rufus*) across three study areas (ESC, CF, and SM), Upper Peninsula of Michigan, USA, 2010–2019. Black lines, shaded area, and *a* represents area convex hull describing isotopic signatures

We estimated dietary niche breadth of 47, 53, and 30 wolves, coyotes, and bobcats, respectively. Increased dietary niche breadth of wolves was not correlated with increased density of wolves (*r* = .29) (Table 1, Figures 2 and 5). However, increased dietary niche breadth of coyotes was correlated with increased coyote density (*r* = .99) and decreased wolf density (*r *= −.71). Increased dietary niche breadth of bobcats was correlated with increased bobcat (*r* = .85) and coyote (*r* = .81) density and decreased wolf density (*r* = −.99).

## 5 DISCUSSION

We found support parallel niche release in increased variability in space use, temporal activity, and dietary niche breadth and density of coyotes corresponding with decreased density of wolves. Increased density of wolves also resulted in increased intraspecific spatial and temporal activity niche breadth. We found limited evidence that varying bobcat niche breadth and density was related to density of coyotes or wolves but identified increased dietary niche breadth with greater bobcat density.

Increased variability in space use of coyotes occurred with decreased wolf and increased coyote density, while increased wolf density was associated with increased variability in space use of wolves. Wolves and coyotes are territorial and space use is influenced by intraspecific density, habitat, and prey distribution (Gese et al., 1988; Laundré & Keller, 1984). High prey density can reduce home range size in both species (Bekoff & Gese, 2003; Mech & Boitani, 2007), and as intraspecific density increases, individuals family groups may be marginalized into areas of lower prey density, requiring increased space use to meet energetic requirements (Mech & Boitani, 2007; Moorcroft et al., 2006). Coyotes exhibit spatial avoidance of wolves and increased wolf density can constrain coyote space use (Arjo & Pletscher, 2000; Berger & Gese, 2007; Flagel et al., 2016), in turn reducing population level variability in space use. Increased variability in bobcat space use in response to lower coyote density may be due to reduced spatial constraints on bobcats (Anderson & Lovallo, 2003). However, density of bobcats in our study varied dramatically which may have been due to greater snow depth in SM (Peers et al., 2013).

Temporal variability in activity of wolves, coyotes, and bobcats increased as wolf density increased and coyote density decreased. Wolves are territorial and increased variation in temporal activity among individuals or packs may be due to increased intraspecific temporal avoidance and patrolling of territories (Mech & Boitani, 2007). Also, decreased density of coyotes may have reduced constraints on bobcat temporal activity, but comparative evidence in the literature is lacking. Greater variability in temporal activity of coyotes and bobcats with increased wolf density could also reflect fine scale temporal avoidance of wolves as observed in coyotes (Petroelje, 2021). As variability in temporal activity for all species increased among study areas with greater human presence, we also suspect reduced risk to humans may have allowed for greater variability in temporal activity patterns (Smith et al., 2018). Future studies may consider a fine‐scale evaluation of factors influencing temporal activity patterns of intraguild carnivores controlling for human induced risk.

Dietary niche breadth and density of coyotes increased with decreasing density of wolves. Dietary niche breadth of bobcats increased with increasing bobcat and wolf density and decreased coyote density. As intraspecific density increases, preferred prey may be less abundant and result in prey switching (Holling, 1959; Solomon, 1949), reported in wolves (Garrott et al., 2007), coyotes (Randa et al., 2009), and bobcats (Blankenship, 2000). Bobcats and coyote exhibit high dietary overlap (Anderson & Lovallo, 2003; Neale & Sacks, 2001) and increased wolf density may have released bobcats from competition with coyotes resulting in greater bobcat dietary breadth. In all species, dietary breadth was positively skewed along the δ^13^C axis in ESC compared with other areas (Figure 5), likely due to scavenging at livestock carcass dumps (Petroelje et al., 2019) or consumption of prey feeding on agricultural crops (i.e., corn [*Zea mays*]) (Bentzen et al., 2014) most prevalent in ESC. Wolf diet was subsidized by livestock carcass dumps in ESC (≥22%, Petroelje et al., 2019); and we suspect isotopic signatures of only wild prey would reduce dietary niche breadth in ESC demonstrating decreased dietary niche breadth with increased density as seen between CF and SM, further supporting parallel niche release for wolves driven by intraspecific competition.

The observed inverse relationship between coyote and wolf densities is supported by other studies in the Greater Yellowstone Ecosystem, USA (GYE) (Berger & Gese, 2007) and Isle Royale National Park, USA (Krefting, 1969), where wolves extirpated coyotes. As in the GYE, habitat heterogeneity in the UP is high and lack of demographic closure will likely allow for sympatric coexistence (Berger & Gese, 2007; Petroelje, 2021). Decreased wolf density among study areas is likely due to increased risk of human‐caused mortality associated with agricultural and developed lands (O'Neil, 2017). Coyotes may select for human modified habitat (Gehrt & McGraw, 2007; Hinton et al., 2015; Van Deelen & Gosselink, 2006) and reduced wolf density and greater human presence may have synergistic benefits for coyotes (Bekhoff & Gese, 2003). Considering humans as competitors with wolves (Hebblewhite et al., 2005), competition between coyotes and wolves may be mediated by humans, and coyotes may be using niche space (Bekhoff & Gese, 2003) largely unavailable to wolves (Mech, 2017). The inverse relationship between coyote and bobcat density in ESC and CF did not support our hypothesis but increased bobcat density may be related to reduced coyote density (Gipson & Kamler, 2002; Henke & Bryant, 1999). Decreased coyote density due to suspected competition with wolves may have released bobcats from competition with coyotes, analogous to the reported wolf–coyote–fox (*Vulpes* spp.) relationship (Levi & Wilmers, 2012; Newsome & Ripple, 2015). However, decreased bobcat density in SM corresponded with the lowest coyote density and greatest wolf density, but greater snow depth in SM may have limited bobcats occurrence (MDNR, unpublished data, Peers et al., 2012). Snow depth alone did not likely limit coyote density in SM (Dowd et al., 2014), and local knowledge suggests coyotes were more abundant before wolf recolonization. The effect of interference competition between wolves and coyotes on bobcat density remains unclear but cumulative evidence suggests little to no effect (Ripple et al., 2013).

Our study supports interference competition between wolves and coyotes (Arjo & Pletscher, 2000; Berger & Gese, 2007; Petroelje, 2021) and demonstrates how increased competition may reduce niche breadth. Coyotes exhibit remarkable ecological plasticity (Bekhoff & Gese, 2003) and their ability to exploit narrow differences in resource availability likely facilitates coexistence with wolves (Petroelje, 2021). Our results also indicate wolves may limit the realized niche of coyotes resulting in decreased coyote density, which supports broader patterns of distribution and abundance (Levi & Wilmers, 2012; Newsome & Ripple, 2015). We found limited support for parallel niche release for bobcats and suspect differences in life history influencing resource use and fundamental niche reduces competition with both wolves and coyotes (Neale & Sacks, 2001).

We found broad support for parallel niche release of subordinate competitors when competition with dominant competitors is relaxed in a large carnivore guild. Increased niche width was correlated with increased density likely indicative of an adaptive advantage to populations. Studies on niche variation have been criticized for reliance on morphological variation which fails to associate with functional variation (Bolnick et al., 2007, but see Lafferty et al., 2015). We estimated variability in niche breadth by the outcomes of animal behavior (suggested by Bolnick et al., 2007) and support parallel niche release through mechanisms driving niche partitioning and constraints on realized niches. This study was limited by our ability to quantify among‐individual variability and simultaneously test the niche variation hypothesis (Van Valen, 1965). Though the variability in competitor density among study areas was dramatic, we were also unable to provide a direct measure of fitness. These shortcoming are due in part to temporal and spatial resource availability and logistics of monitoring large mammals consistently for one or more generations consecutively.

Constraints on realized niches of subordinate species are likely to strengthen as large carnivores continue to recolonize historical range (Garshelis & Hristienko, 2006; LaRue et al., 2012). Increased competition will undoubtedly alter relationships among species (Manlick et al., 2017) and prey population dynamics (Berger & Gese, 2007). Knowledge of the mechanisms which govern competition, and consequently distributions and abundances, is central to our understanding of ecological relationships and for effective management of populations.

## CONFLICT OF INTEREST

The authors declare no conflicts of interest.

## AUTHOR CONTRIBUTIONS

**Nicholas L. Fowler:** Conceptualization (lead); data curation (equal); formal analysis (lead); investigation (equal); methodology (equal); project administration (equal); visualization (equal); writing – original draft (equal); writing – review & editing (equal). **Tyler R. Petroelje:** Data curation (equal); investigation (equal); methodology (equal); project administration (equal); visualization (equal); writing – original draft (equal); writing – review & editing (equal). **Todd M. Kautz:** Data curation (equal); investigation (equal); methodology (equal); project administration (equal); visualization (equal); writing – original draft (equal); writing – review & editing (equal). **Nathan J. Svoboda:** Conceptualization (equal); investigation (equal); methodology (equal); project administration (equal); visualization (equal); writing – original draft (equal); writing – review & editing (equal). **Jared F. Duquette:** Conceptualization (equal); investigation (equal); methodology (equal); project administration (equal); visualization (equal); writing – original draft (equal); writing – review & editing (equal). **Kenneth F. Kellner:** Conceptualization (equal); investigation (equal); methodology (equal); project administration (equal); software (lead); visualization (equal); writing – original draft (equal); writing – review & editing (equal). **Dean E. Beyer Jr:** Conceptualization (equal); funding acquisition (equal); investigation (equal); methodology (equal); project administration (equal); visualization (equal); writing – original draft (equal); writing – review & editing (equal). **Jerrold L. Belant:** Conceptualization (equal); funding acquisition (equal); investigation (equal); methodology (equal); project administration (equal); visualization (equal); writing – original draft (equal); writing – review & editing (equal).

## Supporting information

Supplementary Material

## Data Availability

Animal relocation and stable isotope data are available from Fowler, Nicholas (2022), Relocation Data, Dryad, Dataset, https://doi.org/10.5061/dryad.xsj3tx9gr.
