# Supplementary material for: Variable effects of wolves on niche breadth and density of intraguild competitors
Source: Ecol Evol. 2022 Feb 9;12(2):e8542. doi: 10.1002/ece3.8542 (PMC8829107; doi:10.1002/ece3.8542)
Supplement: Supplementary file 1 — Supplementary Material [file ECE3-12-e8542-s001.docx]

**Supporting Materials**

**Supporting Table 1.** Temporal occurrence of surveys and captures efforts of gray wolves (*Canis lupus*), coyotes (*C. latrans*), and bobcats (*Lynx rufus*) across three study areas, Upper Peninsula of Michigan, USA, 2010–2019.

|  |  |  |  |  |  |
| --- | --- | --- | --- | --- | --- |
| Study Area | Year | Wolf Track Surveys | Coyote Howl Surveys | Bobcat Hair Snares | Carnivore Capture |
| ESC | 2010 | x | x | x | x |
|  | 2011 | x | x | x | x |
| CF | 2012 |  | x |  |  |
|  | 2013 | x | x | x | x |
|  | 2014 | x | x | x | x |
|  | 2015 | x |  | x | x |
| SM | 2016 |  | x |  |  |
|  | 2017 | x | x | x | x |
|  | 2018 | x | x | x | x |
|  | 2019 | x | x | x | x |
|  |  |  |  |  |  |

**Supporting Table 2.** Capture table of gray wolves (*Canis lupus*) (WO), coyotes (*C. latrans*) (CO), and bobcats (*Lynx rufus*) (BC) across three study areas, Upper Peninsula of Michigan, USA, 2010–2019; n = number of locations, First Date = capture date, Last Date = last date collared or considered.

| **Carnivore ID** |  | **n** | **First Date** | **Last Date** | **Days Collared** | **Study Area** | **Sex** |
| --- | --- | --- | --- | --- | --- | --- | --- |
| BC004 |  | 9237 | 6/1/2009 | 8/31/2009 | 91 | ESC | M |
| BC006 |  | 3810 | 6/1/2010 | 6/21/2010 | 20 | ESC | F |
| BC008 |  | 10197 | 6/1/2011 | 8/31/2011 | 91 | ESC | M |
| BC103 |  | 10095 | 6/1/2013 | 8/28/2013 | 88 | CF | F |
| BC104 |  | 8724 | 6/1/2013 | 8/31/2013 | 91 | CF | M |
| BC106 |  | 11399 | 6/1/2014 | 8/31/2014 | 91 | CF | M |
| BC113 |  | 11371 | 6/1/2015 | 8/31/2015 | 91 | CF | M |
| BC300 |  | 10183 | 6/1/2017 | 8/31/2017 | 91 | SM | M |
| BC301 |  | 10152 | 6/1/2017 | 8/31/2017 | 91 | SM | F |
| BC304 |  | 7506 | 6/14/2019 | 8/31/2019 | 78 | SM | M |
| CO001 |  | 6900 | 6/1/2009 | 8/31/2009 | 91 | ESC | F |
| CO002 |  | 6916 | 6/1/2009 | 8/31/2009 | 91 | ESC | F |
| CO004 |  | 9023 | 6/1/2009 | 8/31/2009 | 91 | ESC | M |
| CO005 |  | 8997 | 6/1/2009 | 8/31/2009 | 91 | ESC | F |
| CO006 |  | 8867 | 6/1/2009 | 8/31/2009 | 91 | ESC | F |
| CO007 |  | 8735 | 6/1/2009 | 8/31/2009 | 91 | ESC | M |
| CO008 |  | 8141 | 6/5/2009 | 8/31/2009 | 87 | ESC | F |
| CO009 |  | 6987 | 6/10/2009 | 8/31/2009 | 82 | ESC | F |
| CO010 |  | 10871 | 6/1/2010 | 8/31/2010 | 91 | ESC | M |
| CO011 |  | 11408 | 6/1/2010 | 8/31/2010 | 91 | ESC | M |
| CO015 |  | 10170 | 6/1/2010 | 8/31/2010 | 91 | ESC | F |
| CO016 |  | 9474 | 6/1/2010 | 8/31/2010 | 91 | ESC | M |
| CO017 |  | 8062 | 6/8/2010 | 8/31/2010 | 84 | ESC | F |
| CO020 |  | 7843 | 6/10/2010 | 8/31/2010 | 82 | ESC | M |
| CO023 |  | 10960 | 6/1/2011 | 8/31/2011 | 91 | ESC | M |
| CO024 |  | 11633 | 6/1/2011 | 8/31/2011 | 91 | ESC | F |
| CO025 |  | 10889 | 6/1/2011 | 8/26/2011 | 86 | ESC | M |
| CO026 |  | 10258 | 6/1/2011 | 8/31/2011 | 91 | ESC | F |
| CO027 |  | 9438 | 6/1/2011 | 8/31/2011 | 91 | ESC | M |
| CO029 |  | 7810 | 6/1/2011 | 8/31/2011 | 91 | ESC | M |
| CO102 |  | 10280 | 6/1/2013 | 8/27/2013 | 87 | CF | F |
| CO104 |  | 9140 | 6/1/2013 | 8/31/2013 | 91 | CF | F |
| CO105 |  | 9074 | 6/1/2013 | 8/31/2013 | 91 | CF | F |
| CO107 |  | 7397 | 6/15/2013 | 8/31/2013 | 77 | CF | M |
| CO110 |  | 8399 | 6/5/2014 | 8/31/2014 | 87 | CF | F |
| CO111 |  | 7750 | 6/9/2014 | 8/31/2014 | 83 | CF | F |
| CO112 |  | 5013 | 6/26/2014 | 8/31/2014 | 66 | CF | F |
| CO113 |  | 11573 | 6/1/2015 | 8/31/2015 | 91 | CF | F |
| CO114 |  | 9742 | 6/1/2015 | 8/22/2015 | 82 | CF | F |
| CO115 |  | 9794 | 6/1/2015 | 8/31/2015 | 91 | CF | M |
| CO119 |  | 3188 | 6/10/2015 | 7/13/2015 | 33 | CF | F |
| CO300 |  | 8479 | 6/3/2017 | 8/31/2017 | 89 | SM | M |
| CO301 |  | 1822 | 8/7/2017 | 8/29/2017 | 22 | SM | F |
| CO302 |  | 7063 | 6/19/2018 | 8/31/2018 | 73 | SM | M |
| CO304 |  | 1430 | 6/1/2019 | 6/12/2019 | 11 | SM | F |
| WO001 |  | 6606 | 6/1/2009 | 8/31/2009 | 91 | ESC | F |
| WO002 |  | 6776 | 6/1/2009 | 8/31/2009 | 91 | ESC | M |
| WO006 |  | 10337 | 6/1/2010 | 8/31/2010 | 91 | ESC | M |
| WO007 |  | 9532 | 6/1/2010 | 8/31/2010 | 91 | ESC | F |
| WO008 |  | 10129 | 6/1/2011 | 8/25/2011 | 85 | ESC | F |
| WO010 |  | 8666 | 6/1/2011 | 8/31/2011 | 91 | ESC | F |
| WO101 |  | 8863 | 6/1/2013 | 8/31/2013 | 91 | CF | M |
| WO102 |  | 6857 | 6/6/2013 | 8/22/2013 | 77 | CF | M |
| WO103 |  | 8086 | 6/7/2013 | 8/31/2013 | 85 | CF | F |
| WO104 |  | 7496 | 6/12/2013 | 8/31/2013 | 80 | CF | M |
| WO105 |  | 10269 | 6/1/2014 | 8/31/2014 | 91 | CF | F |
| WO106 |  | 9499 | 6/1/2014 | 8/31/2014 | 91 | CF | M |
| WO107 |  | 7071 | 6/13/2014 | 8/31/2014 | 79 | CF | F |
| WO110 |  | 10860 | 6/1/2015 | 8/31/2015 | 91 | CF | F |
| WO112 |  | 4309 | 6/3/2015 | 7/20/2015 | 47 | CF | F |
| WO301 |  | 3258 | 6/1/2017 | 6/19/2017 | 18 | SM | M |
| WO303 |  | 9499 | 6/1/2017 | 8/31/2017 | 91 | SM | M |
| WO304 |  | 8893 | 6/1/2017 | 8/31/2017 | 91 | SM | M |
| WO306 |  | 8288 | 6/3/2017 | 8/31/2017 | 89 | SM | M |
| WO307 |  | 8097 | 6/7/2017 | 8/31/2017 | 85 | SM | F |
| WO308 |  | 2965 | 6/1/2018 | 8/31/2018 | 91 | SM | F |
| WO309 |  | 7042 | 6/1/2018 | 8/31/2018 | 91 | SM | M |
| WO311 |  | 6170 | 6/2/2018 | 8/31/2018 | 90 | SM | F |
| WO312 |  | 6395 | 6/8/2018 | 8/31/2018 | 84 | SM | F |
| WO313 |  | 7123 | 6/8/2018 | 8/31/2018 | 84 | SM | M |
| WO314 |  | 5562 | 6/16/2018 | 8/31/2018 | 76 | SM | F |
| WO316 |  | 6197 | 6/27/2018 | 8/31/2018 | 65 | SM | M |
| WO317 |  | 8156 | 6/1/2019 | 8/14/2019 | 74 | SM | F |
| WO318 |  | 8431 | 6/1/2019 | 8/31/2019 | 91 | SM | M |
| WO319 |  | 3696 | 6/1/2019 | 7/22/2019 | 51 | SM | M |
| WO321 |  | 7357 | 6/1/2019 | 8/23/2019 | 83 | SM | M |
| WO322 |  | 5027 | 6/1/2019 | 8/1/2019 | 61 | SM | M |
| WO323 |  | 7266 | 6/1/2019 | 8/31/2019 | 91 | SM | F |

**Supporting Table 3.** Gray wolf (*Canis lupus*) (WO), coyote (*C. latrans*) (CO), and bobcat (*Lynx rufus*) (BC) core home range area (June–August) across 3 study areas, Upper Peninsula of Michigan, USA, 2010–2019.

| **Carnivore ID** | **Area (km^2^)** | **Year Collared** | **Study Area** |
| --- | --- | --- | --- |
| BC004 | 136.77 | 2009 | ESC |
| BC006 | 5.24 | 2010 | ESC |
| BC008 | 82.47 | 2011 | ESC |
| BC103 | 11.33 | 2013 | CF |
| BC104 | 12.06 | 2013 | CF |
| BC106 | 79.22 | 2014 | CF |
| BC106 | 92.73 | 2015 | CF |
| BC111 | 4.54 | 2015 | CF |
| BC113 | 25.74 | 2015 | CF |
| BC300 | 78.59 | 2017 | SM |
| BC301 | 136.44 | 2017 | SM |
| BC301 | 85.50 | 2018 | SM |
| BC304 | 185.76 | 2019 | SM |
| CO001 | 48.58 | 2009 | ESC |
| CO002 | 3.10 | 2009 | ESC |
| CO004 | 3.71 | 2009 | ESC |
| CO005 | 2.98 | 2009 | ESC |
| CO006 | 12.30 | 2009 | ESC |
| CO007 | 2.97 | 2009 | ESC |
| CO008 | 86.71 | 2009 | ESC |
| CO009 | 1.82 | 2009 | ESC |
| CO010 | 4.48 | 2010 | ESC |
| CO011 | 34.75 | 2010 | ESC |
| CO012 | 3.13 | 2010 | ESC |
| CO015 | 135.11 | 2010 | ESC |
| CO016 | 109.60 | 2010 | ESC |
| CO017 | 127.10 | 2010 | ESC |
| CO017 | 143.24 | 2011 | ESC |
| CO020 | 7.89 | 2010 | ESC |
| CO023 | 2.20 | 2011 | ESC |
| CO024 | 2.34 | 2011 | ESC |
| CO025 | 4.65 | 2011 | ESC |
| CO026 | 10.55 | 2011 | ESC |
| CO027 | 4.01 | 2011 | ESC |
| CO029 | 59.46 | 2011 | ESC |
| CO102 | 6.31 | 2013 | CF |
| CO104 | 40.84 | 2013 | CF |
| CO105 | 122.76 | 2013 | CF |
| CO107 | 3.84 | 2013 | CF |
| CO110 | 52.03 | 2014 | CF |
| CO111 | 2.17 | 2014 | CF |
| CO112 | 6.32 | 2014 | CF |
| CO113 | 3.64 | 2015 | CF |
| CO114 | 1.94 | 2015 | CF |
| CO115 | 12.73 | 2015 | CF |
| CO119 | 5.56 | 2015 | CF |
| CO300 | 7.58 | 2017 | SM |
| CO301 | 15.48 | 2017 | SM |
| CO302 | 6.77 | 2018 | SM |
| CO304 | 12.88 | 2019 | SM |
| WO001 | 22.96 | 2009 | ESC |
| WO002 | 10.06 | 2009 | ESC |
| WO006 | 7.62 | 2010 | ESC |
| WO007 | 20.7 | 2010 | ESC |
| WO008 | 22.35 | 2011 | ESC |
| WO010 | 17.62 | 2011 | ESC |
| WO101 | 24.5 | 2013 | CF |
| WO102 | 43.47 | 2013 | CF |
| WO103 | 24.28 | 2013 | CF |
| WO104 | 30.74 | 2013 | CF |
| WO105 | 72.59 | 2014 | CF |
| WO106 | 45.51 | 2014 | CF |
| WO107 | 15.87 | 2014 | CF |
| WO110 | 50.53 | 2015 | CF |
| WO112 | 49.57 | 2015 | CF |
| WO301 | 13.3 | 2017 | SM |
| WO303 | 28.11 | 2017 | SM |
| WO304 | 68.17 | 2017 | SM |
| WO305 | 97.00 | 2017 | SM |
| WO306 | 77.53 | 2017 | SM |
| WO307 | 59.64 | 2017 | SM |
| WO308 | 67.86 | 2018 | SM |
| WO309 | 30.24 | 2018 | SM |
| WO311 | 42.01 | 2018 | SM |
| WO311 | 40.75 | 2019 | SM |
| WO312 | 29.06 | 2018 | SM |
| WO312 | 1.71 | 2019 | SM |
| WO313 | 31.69 | 2018 | SM |
| WO314 | 24.89 | 2018 | SM |
| WO314 | 58.07 | 2019 | SM |
| WO316 | 18.50 | 2018 | SM |
| WO317 | 33.89 | 2019 | SM |
| WO318 | 32.81 | 2019 | SM |
| WO319 | 26.32 | 2019 | SM |
| WO320 | 33.86 | 2019 | SM |
| WO321 | 82.93 | 2019 | SM |
| WO322 | 20.55 | 2019 | SM |
| WO323 | 34.09 | 2019 | SM |

**S Table 4.** Results of Bartlett’s K-squared test for homogeneity of hourly variance of velocity (meters per second) for gray wolves (*Canis* *lupus*), coyotes (*C. latrans*), and bobcat (*Lynx* *rufus*) across three study areas, Upper Peninsula of Michigan, USA, 2010–2019. Tests were performed with one-tail (α = 0.05) for increased variance corresponding with increased wolf density.

|  | Wolves | | | | | | Coyotes | | | | | | Bobcats | | | | | |
| --- | --- | --- | --- | --- | --- | --- | --- | --- | --- | --- | --- | --- | --- | --- | --- | --- | --- | --- |
|  | Study Area | | | | | | | | | | | | | | | | | |
|  | ESC - CF | | CF - SM | | ESC - SM | | ESC - CF | | CF - SM | | ESC - SM | | ESC - CF | | CF - SM | | ESC - SM | |
| Hour | B-K | p-value | B-K | p-value | B-K | p-value | B-K | p-value | B-K | p-value | B-K | p-value | B-K | p-value | B-K | p-value | B-K | p-value |
| 0 | 0.99 | 0.32 | 1.92 | 0.17 | 4.06 | 0.04 | 0.21 | 0.65 | 3.98 | 0.05 | 7.27 | 0.01 | 3.25 | 0.07 | 0.36 | 0.55 | 4.05 | 0.04 |
| 1 | 4.29 | 0.04 | 0.84 | 0.36 | 7.2 | 0.01 | 0.52 | 0.47 | 5.37 | 0.02 | 11.14 | 0.00 | 3.97 | 0.05 | 3.06 | 0.08 | 6.82 | 0.01 |
| 2 | 0.06 | 0.8 | 1.18 | 0.28 | 1.26 | 0.26 | 0.04 | 0.84 | 5.09 | 0.02 | 7.94 | 0.00 | 3.31 | 0.07 | 3.26 | 0.07 | 6.25 | 0.01 |
| 3 | 0.43 | 0.51 | 0.01 | 0.93 | 0.64 | 0.42 | 0.38 | 0.54 | 6.82 | 0.01 | 6.65 | 0.01 | 4.35 | 0.04 | 1.51 | 0.22 | 6.24 | 0.01 |
| 4 | 0.00 | 0.99 | 3.84 | 0.05 | 2.49 | 0.11 | 0.76 | 0.38 | 5.56 | 0.02 | 12.39 | 0.00 | 4.3 | 0.04 | 3.74 | 0.05 | 7.52 | 0.01 |
| 5 | 0.00 | 0.98 | 1.95 | 0.16 | 1.22 | 0.27 | 1.03 | 0.31 | 6.29 | 0.01 | 4.37 | 0.04 | 3.96 | 0.05 | 2.13 | 0.14 | 6.27 | 0.01 |
| 6 | 0.25 | 0.62 | 2.71 | 0.10 | 0.73 | 0.39 | 1.56 | 0.21 | 7.88 | 0.00 | 5.13 | 0.02 | 1.8 | 0.18 | 2.16 | 0.14 | 4.02 | 0.04 |
| 7 | 0.08 | 0.78 | 3.25 | 0.07 | 1.44 | 0.23 | 4.52 | 0.03 | 10.86 | 0.00 | 4.27 | 0.04 | 1.03 | 0.31 | 0.86 | 0.35 | 2.22 | 0.14 |
| 8 | 1.42 | 0.23 | 0.22 | 0.64 | 2.57 | 0.11 | 1.09 | 0.30 | 7.35 | 0.01 | 5.41 | 0.02 | 0.91 | 0.34 | 0.45 | 0.5 | 1.70 | 0.19 |
| 9 | 0.13 | 0.72 | 0.50 | 0.48 | 0.90 | 0.34 | 0.37 | 0.54 | 1.74 | 0.19 | 3.86 | 0.05 | 0.58 | 0.45 | 1.36 | 0.24 | 2.01 | 0.16 |
| 10 | 0.04 | 0.83 | 0.00 | 0.95 | 0.04 | 0.85 | 0.00 | 0.95 | 3.01 | 0.08 | 3.74 | 0.05 | 0.24 | 0.63 | 1.42 | 0.23 | 1.48 | 0.22 |
| 11 | 0.64 | 0.42 | 0.04 | 0.85 | 0.57 | 0.45 | 0.14 | 0.71 | 2.60 | 0.11 | 2.36 | 0.12 | 0.66 | 0.41 | 0.66 | 0.42 | 0.01 | 0.93 |
| 12 | 0.02 | 0.88 | 0.20 | 0.66 | 0.27 | 0.6 | 0.26 | 0.61 | 1.18 | 0.28 | 0.68 | 0.41 | 0.16 | 0.69 | 0.53 | 0.47 | 0.04 | 0.84 |
| 13 | 0.12 | 0.73 | 0.52 | 0.47 | 0.89 | 0.35 | 1.16 | 0.28 | 2.41 | 0.12 | 0.82 | 0.37 | 0.02 | 0.88 | 0.25 | 0.62 | 0.25 | 0.62 |
| 14 | 0.05 | 0.82 | 16.09 | 0.00 | 9.23 | 0.00 | 0.03 | 0.87 | 0.24 | 0.62 | 0.18 | 0.67 | 0.05 | 0.81 | 1.79 | 0.18 | 0.58 | 0.45 |
| 15 | 0.00 | 0.99 | 2.71 | 0.10 | 1.75 | 0.19 | 0.25 | 0.61 | 0.88 | 0.35 | 0.45 | 0.5 | 0.51 | 0.48 | 1.15 | 0.28 | 0.04 | 0.85 |
| 16 | 0.02 | 0.88 | 2.02 | 0.16 | 1.67 | 0.20 | 1.90 | 0.17 | 6.08 | 0.01 | 2.92 | 0.09 | 0.00 | 0.96 | 1.13 | 0.29 | 0.65 | 0.42 |
| 17 | 0.57 | 0.45 | 0.26 | 0.61 | 1.45 | 0.23 | 0.53 | 0.47 | 0.89 | 0.35 | 0.26 | 0.61 | 0.10 | 0.75 | 2.07 | 0.15 | 0.59 | 0.44 |
| 18 | 3.45 | 0.06 | 0.10 | 0.75 | 4.69 | 0.03 | 0.47 | 0.49 | 5.04 | 0.02 | 4.17 | 0.04 | 0.3 | 0.58 | 0.00 | 0.96 | 0.22 | 0.64 |
| 19 | 5.74 | 0.02 | 3.93 | 0.05 | 2.26 | 0.13 | 0.74 | 0.39 | 3.92 | 0.05 | 2.48 | 0.12 | 0.26 | 0.61 | 0.01 | 0.9 | 0.15 | 0.70 |
| 20 | 2.69 | 0.1 | 3.84 | 0.05 | 0.3 | 0.59 | 1.09 | 0.3 | 0.54 | 0.46 | 0.01 | 0.93 | 0.62 | 0.43 | 0.01 | 0.91 | 0.43 | 0.51 |
| 21 | 0.35 | 0.56 | 0.07 | 0.79 | 0.21 | 0.64 | 0.1 | 0.76 | 4.88 | 0.03 | 8.11 | 0 | 0.67 | 0.41 | 0.73 | 0.39 | 1.67 | 0.2 |
| 22 | 0.03 | 0.86 | 1.19 | 0.28 | 1.12 | 0.29 | 0.94 | 0.33 | 1.14 | 0.29 | 3.72 | 0.05 | 8.19 | 0 | 0.4 | 0.53 | 8.98 | 0 |
| 23 | 0.7 | 0.4 | 0.97 | 0.32 | 2.57 | 0.11 | 0.16 | 0.68 | 3.21 | 0.07 | 5.72 | 0.02 | 4.77 | 0.03 | 0.02 | 0.88 | 4.77 | 0.03 |

**S Results 1**

*Population Estimation*

- Mean number of wolves per pack was 5.50 (sd = 1.12), 5.64 (sd = 1.87), and 5.30 (sd = 1.43) in ESC, CF, and SM, respectively.
- Mean number of coyote responses detected across years was 249.50 (sd = 28.50), 170.67 (sd = 24.85), and 76.50 (sd = 13.05) in ESC, CF, and SM, respectively. Mean percentage of sites with > 1 detection across years was 80.00, 82.50, and 47.50% in ESC, CF, and SM, respectively. Estimated mean detection probability was 0.06, 0.11, and 0.13 in ESC, CF, and SM, respectively. Estimated coyote density was 32.68 (12.66–207.54 confidence interval [CI]), 6.16 (3.24–36.57 CI), and 2.57 (1.132–8.32 CI) individuals per 100 km^2^ in ESC, CF, and SM, respectively (Figure 2).
- Across years, we collected 34, 62, and 44 hair samples identified as bobcat with sufficient quality to genotype individuals in ESC, CF, and SM, respectively. We detected 16, 29, and 2 individuals in ESC, CF, and SM, respectively. Number of detections per individual in ESC, CF, and SM was 1–4, 1–5, and 1–5, respectively. Estimated bobcat density was 2.95 (0.91–2.99 CI), 3.8 (2.6–4.9 CI), individuals per 100 km^2^ in ESC and CF, respectively (Figure 2). Extreme winter weather limited the final SM survey to 6 sampling occasions. Due to few detections across years, we were unable to estimate bobcat density in SM. We report density as the number of individuals detected through hair snares and capture efforts across years at 7 individuals (0.32 per 100 km^2^, Figure 2).

*Home Range Estimation*

- Across study areas, mean core UD areas were 40.06 km^2^ (sd = 25.76) for wolves, 30.04 km^2^ (sd = 43.74) for coyotes, and 72.03 km^2^ (sd = 58.12) for bobcats (S Table 3).

*Activity Pattern*

- See S Table 4

*Stable Isotope Analysis*

- We estimated isotopic niche breadth of 47, 53, and 30 wolves, coyotes, and bobcats, respectively. Estimated isotopic niche breadth of wolves was greatest in ESC (3.19) followed by SM (1.13) and CF (3.14) (Figure 5).
- For coyotes there was a 53.57 and 9.05% decrease in isotopic niche breadth from ESC (9.52) to CF (4.4) and CF to SM (4.02), respectively (Figure 5).
- In bobcats there was an observed 19.16 and 53.36% decrease in isotopic niche breadth from ESC (3.13) to CF (2.52) and CF to SM (1.18), respectively (Figure 5).
